# Significant gender-related differences in CVD have been recognised only recently: deficits in female healthcare

**DOI:** 10.1186/1878-5085-5-S1-A88

**Published:** 2014-02-11

**Authors:** Olga Golubnitschaja

**Affiliations:** 1 Molecular Diagnostics, Radiological Clinic, Medical Faculty, Friedrich-Wilhelms-University of Bonn, Germany

## 

Evidence-based publications indicating significant gender-related differences in CVD diagnosis, manifestation and treatments as well as particular problems shifted to the female subpopulations appeared quite recently [1]. Although CVD is traditionally considered as the major health burden of male subpopulations surprising statistics have been collected for worsening the situation in females. New trends obviously demonstrate a shifting of the problem into healthcare of female populations: from 25 countries selected by World Health Organisation in the years 1990-2000, more than a half (15 countries, 60 %) currently demonstrate either increase in CVD-related death in females or, even if the rates are decreased then less compared to men [1]:

1^st^ group: increased CVD-related death in females

❖ Ukraine – 63%

❖ Kazakhstan – 58%

❖ Belarus – 48 %

❖ Russian Federation – 33%

❖ Romania – 20%

❖ Japan – 13%

❖ Mexico – 2%

2^nd^ group: CVD-related death is less decreased in females versus males

❖ Norway – 17% less

❖ Germany – 4% less

❖ Hungary – 4% less

❖ Luxemburg – 4% less

❖ USA – 4% less

❖ Italy – 2% less

❖ Portugal – 2% less

❖ Armenia – 1% less

Further, current reports (as summarised in [1]) indicate that

• women experience longer stays in hospital related to CVD and suffer greater complications and disability than men: negative social and economical impacts

• diagnostic approaches have been created for men but not adapted for females

• Women experience delayed care in the case of CVD emergency

• Women with acute myocardial infarction have higher rates of mortality than men of the same age

• Short-term mortality after coronary intervention is higher in women than in men

• Although the coincidence of CVD with other fatal diseases is common in women, consistent data do not exist to recognise co-morbidities by patient profiling.

In this context it becomes clear, why women have been reported to consume more hospital resources than men [2]. Those statistics have been collected for CVD in general but, the particular significance is reported for diagnosed atrial fibrillation, heart failure and acute myocardial infarction [1,2]. Hence, the American National Hospital Discharge Survey reports stable rates of hospital admission for men *versus* permanently increasing ones for the age-matched women with diagnosed heart failure [3]: the female gender-specific increase comprises about 19% in North America since 1990. Further, high risk of stroke as the secondary complication is the gender particularity acknowledged in women diagnosed with atrial fibrillation, in contrast to none of the predisposition as observed in men [4]. Delayed recognition of the mounting problems creates a consequent delay in covering multifaceted knowledge deficits accumulated in CVD-related female healthcare which societies around the world are currently facing. Consequently there is an urgent need to elaborate the targeted measures forcing the improvement. The recommendations might be proposed as follows:

1. identify deficits that altogether have resulted in current unsatisfactory situation;

2. analyse rapidly worsening versus improving regional healthcare situations and identify the most specific particularities of both;

3. elaborate effective strategies for covering the recognised deficits at all levels of stakeholders that should be obligatory involved in the process of reorganisation including applicative science, social, economical and political levels;

4. consider to force the practical implementation of innovative approaches in predictive diagnostics and targeted prevention that are rapidly developing in pre-clinical trials;

5. create/ readdress sufficient budgets for educational measures, training purposes and staffing the experts essentially involved in the field.
